# Immunotherapy in Adolescents and Young Adults: What Remains in Cancer Survivors?

**DOI:** 10.3389/fonc.2021.736123

**Published:** 2021-09-23

**Authors:** Enrica Teresa Tanda, Elena Croce, Francesco Spagnolo, Lodovica Zullo, Stefano Spinaci, Carlo Genova, Giovanni Rossi

**Affiliations:** ^1^ Istituto di Ricovero e Cura A Carattere Scientifico (IRCCS) Ospedale Policlinico San Martino-Oncologia Medica 2, Genova, Italy; ^2^ Genetics of Rare Cancers, Department of Internal Medicine and Medical Specialties, University of Genoa, Genova, Italy; ^3^ Division of Breast Surgery, Ospedale Villa Scassi, Genova, Italy; ^4^ UO Clinica di Oncologia Medica, IRCCS Ospedale Policlinico San Martino, Genoa, Italy; ^5^ Department of Internal Medicine and Medical Specialties (DIMI), Università degli Studi di Genova, Genoa, Italy; ^6^ Medical Oncology Department, Ospedale Padre Antero Micone, Genoa, Italy; ^7^ Department of Medical, Surgical and Experimental Sciences, University of Sassari, Sassari, Italy

**Keywords:** AYA, adolescent, young adults, immunotherapy, pregnancy, fertility, long-term toxicities, checkpoint inhibitors immunotherapy and AYA patients

## Abstract

Immunotherapy has changed the landscape of treatments for advanced disease in multiple neoplasms. More and more patients are long survivors from a metastatic disease. Most recently, the extension of indications and evidence of efficacy in early disease settings, such as the adjuvant and neoadjuvant setting in breast cancer, lung cancer, glioma, and gastric cancer, places more attention on what happens to patients who survive cancer. In particular, we evaluated what happens in young patients, a population in whom some immune-related effects are still poorly described. Immunotherapy is already a reality in early disease settings and the scientific community is lagging in describing what to expect in adolescent and young adult (AYA) patients. For instance, the impact of these therapies on female and male fertility is not clear, similarly to the interaction that may occur between these drugs and pregnancy. This review aims to highlight these little-known topics that are difficult to evaluate in *ad hoc* studies.

## Introduction

Immunotherapy has changed contemporary oncology by significantly modifying the outcomes of patients with metastatic cancer. These advances have led to trials evaluating the use of immunotherapy in the earliest settings. First, in advanced diseases, we began to see long survivors; now, thanks to immunotherapy, we expect to see more and more patients who can be defined as cured after a diagnosis of cancer. Although the side effects related to immunotherapy are now widely known, there are still areas of uncertainty. In particular, the possible side effects of immunotherapy specific to the population of adolescents and young adults [AYA aged 15–39 (1)] are currently little explored. Traditionally, these tumors have not received the attention reserved for tumors of pediatric age or adulthood. Generally poorly considered, they are configured as a group of pathologies with a dismal prognosis (2), with a different epidemiological, genetic, molecular, and therapeutic profile compared to what can be observed in other most studied age groups. The explanation for their poor prognosis basically lies in three factors.

1. Delay in diagnosis. It is very difficult to hypothesize a neoplastic disease in the first place in this portion of the population, and the diagnosis is often delayed due to “more common” pathologies being initially considered during the differential diagnosis workup for that age group.
2. Different molecular profile. A growing body of evidence indicates that cancers in AYAs differ in molecular terms from those of pediatric and older age groups. This difference could probably explain the different etiopathogenesis, aggressiveness, and, consequently, the different response to treatments.
3. This is an under-represented population in clinical trials, and there are very few data. A meta-analysis showed that out of 2,176 trials analyzed, only 5 trials were AYA-specific. The 18-year limit imposed by most studies greatly limits access to clinical trials and thus to innovative therapies for the AYA patient group (3).

The introduction of immunotherapy in earlier settings (**Table 1**) and with more extensive indications is exposing an increasing number of young patients to these new drugs. Furthermore, this population can face side effects that in the elderly patient are not evaluable or that implicate a slight impact in their daily life (such as the impact on fertility and/or pregnancy).

**Table 1:** Published trials of immunotherapy in early stage on most common AYAs neoplasms.

Neoplasm	Trial	Phase	Setting	Pts	Results
Melanoma	Opacin-Neo	II	Neoadiuvant	89	pR 80% (arm A), 77% (arm B), 65% (arm C)
Gliomas	Cloughesy et al.	I-II	Neoadjuvant *vs*. adjuvant	30	PFS: 77.5 days *vs*. 99.5 days, HR 0.43 (*p* = 0.03)
Breast	Keynote 522	III	Neoadjuvant	1174	pCR 64.8% *vs*. 51.2%
Breast	IMpassion 031	III	Neoadjuvant	333	pCR 58% *vs*. 41%

pR, pathologic response; PFS, progression-free survival; pCR, pathological complete response.

The aim of this paper is to evaluate the impact of immunotherapy on the subgroup of young patients, evaluating efficacy and any side effects, including the ones that may occur in the long term and/or are more specifically related to this age.

## Epidemiology

In the US, about 89,500 cases of tumors are estimated for the year 2020 in the AYA group, with about 9,270 deaths (1). In the last decade, incidence rates have shown an increasing trend, mainly driven by the increase of thyroid cancers incidence. Five-year survival increased compared to that recorded in the 1970s. However, even in this case, the positive result is heavily influenced by the excellent prognosis of thyroid cancer, testicular cancer, Hodgkin’s lymphoma, and melanoma at early stage.

According to data from Global cancer observatory (4), in 2020, approximately 159,300 AYAs received cancer diagnoses in Europe. Considering both sexes and solid tumors only (thus excluding leukemia and lymphoma), the most diagnosed tumors in AYAs are breast cancer, thyroid cancer, and melanoma. Excluding thyroid cancer, whose rapid increase in incidence seems to be substantially due to an overdiagnosis of very early forms, the third place for incidence is occupied by testicular cancer. Among men, the three most diagnosed cancers are testicular cancer, melanoma, and central nervous system (CNS) cancers. Among women, breast cancer, cervical cancer, and melanoma are the most represented solid tumor diagnoses. Cancer-related mortality in this segment of the population is mostly due to tumors of the CNS, colorectal cancer (CRC), and lung cancer for men; breast cancer, cervical cancer, and CNS tumors are the most frequent cancer-related cause of death for young women.

Furthermore, within the AYAs, the incidence of different neoplasms tends to vary as we get closer to adulthood. For example, breast cancer melanoma and testicular cancer show an increasing incidence as the age gets higher, whereas in the purely adolescent age group, hematological and CNS tumors prevail (1).

### Brain Tumors

Estimation of new diagnoses of brain tumors in 2020 predicts about 7,648 new cases in the US with a crude rate of 2.0 per 100,000) and, similarly, 8,618 new cases in Europe with a crude rate of 2.9 per 100,000 in AYA patients (4). An epidemiological explanation for this small and non-significant difference in the crude rate between the EU and the US is currently not available. Gliomas seem to cover most of the diagnoses, as in other age groups, but with some age-related differences: indeed, adolescents have higher incidence of brain tumors strictly related to childhood (e.g., medulloblastoma). Five-year survival ranges from 77% in adolescents to 66% in patients 30 to 39 years of age, reflecting the difference in aggressiveness in glioma subtypes.

Recently, some promising results on the activity of nivolumab in the neoadjuvant setting of glioblastoma in patients with a mean age of 55.4 ± 13.5 years old have been released (5) (**Table 1**). A phase 2 trial, including patients between 6 months and 22 years old, is currently ongoing, with the aim to confirm these preliminary data (6).

### Breast Cancer

We expect about 39,526 new cases in the European Union (EU) alone. The risk of developing breast cancer at a young age increases in case of family history, or presence of BRCA 1/BRCA 2 mutation or other genetic syndromes (7–9). Over the past 10 years, the incidence of breast cancer remained stable in adolescents, increased by about 2% per year in patients aged 20 to 30, and by 0.2% per year in patients aged between 30 and 39. Factors that may have driven this increase in incidence include new reproductive habits (later pregnancies). AYA patients are often diagnosed later than older women and are diagnosed with infiltrative rather than *in situ* neoplasm, three times more common in screened patients (10). The explanation lies both in the fact that this is a population not subjected to mammography screening and in the higher incidence of more aggressive tumors in this population [triple-negative breast cancers (TNBCs)] (11–13). As a result, the 5-year survival of these patients is significantly lower than in more advanced age groups (86% *vs*. 91% in the 45- to 64-year-old group) (14).

Recently, immunotherapy has achieved a higher rate of pathological complete response than chemotherapy alone, in a neoadjuvant setting. In particular, Keynote522 tested pembrolizumab plus carboplatin and paclitaxel followed by Anthracycline and cyclophosphamide in early-stage TNBCs (15). The study included patients who were at least 18 years old, with a median age of 48 in the placebo group and 49 in the pembrolizumab group. The study demonstrated 64.8% *vs*. 51.2% of pathological complete response (CR) in the pembrolizumab plus chemotherapy group *versus* the chemotherapy-alone group. Similarly, in 2020, results from another neoadjuvant trial, IMpassion 031, evaluating atezolizumab in the experimental arm, were published. The study included patients aged 18 years or older, with a median age of 51 in both the atezolizumab plus chemotherapy group and the placebo plus chemotherapy group. The rate of pathological CR was 58% in the experimental arm and 41% in the control arm. These two trials will soon change clinical practice in early breast cancer (**Table 1**). More patients will be cured thanks to immunotherapy, but data about long-term toxicity are still to be studied.

### Melanoma

In 2020 16,769 melanoma cases were estimated in EU in the AYA age group.

The incidence of melanoma among adolescents is decreasing, while it remains stable in the 30- to 39-year-old group. The reason seems to be attributable to the greater dissuasion from the use of sun beds and the increase in the use of healthy and controlled behaviors regarding sun exposure (16). At the same time, mortality rapidly declined, reaching 5% per year, with a 5-year survival exceeding 94%. However, this result is probably driven by early diagnosis.

In the AYA subgroup of patients, melanoma seems to affect women more than men (17), in contrast to what is observed in elder age groups.

Generally, young patients develop melanoma due to the combination of genetic substrate and exposure to UV rays. The risk factors that are most associated with melanoma at a young age include the presence of congenital giant melanocytic nevi, xeroderma pigmentosum, dysplastic nevus syndrome, and immunosuppression (18). The use of sun beds has globally decreased in Europe throughout the last years (19, 20), but it remains an important risk factor among young women. The use of sun beds appears to play a particularly important role in the AYA subgroup, indeed, and the risk of developing melanoma is about 60% higher in people who started using sun beds at the age of less than 35. Moreover, the risk increases with the duration and intensity of exposure (21, 22).

The clinical diagnosis of melanoma in this subgroup of patients can be particularly difficult, since other benign pigmented lesions that share some characteristics with melanomas are quite frequent in this population. Histologically, a large portion of these melanomas is represented by spitzoid melanomas, which are often difficult to distinguish from atypical spitz tumors (23).

The melanoma of the AYA group has not only different histopathological characteristics, but also a different clinical behavior compared to melanoma observed in the adult population. The prognosis and survival rate seem to be comparable to the lesions of adulthood, but the thickness of the lesions tends to be higher, the lymph node invasion more frequent, whereas they tend to relapse at a distance with less frequency (24–26). Most of these patients (>80%) present in stage I–II, while 10.15% present in stage III and 1%–3% present in stage IV. Considering the genotype, AYA group melanomas exhibit high levels of MSH (MutS Homolog) compared to adult melanomas, in addition to a *BRAF* mutation frequency rate of about 90% (27, 28). The higher frequency of *BRAF* mutation in young patients has not yet be explained (29).

### Tumors of the Testis

There are 16,552 new estimated cases in the US and 16,255 new estimated cases in the EU for the year 2020 (4). Over the past 10 years, the incidence rate has grown from 0.4% to 1.1% annually, while the mortality rate remained stable. Five-year survival exceeds 95%. To date, no data about the efficacy of immunotherapy in germ-cell tumors are available.

### Cervical Cancer

For 2020, about 15,188 new cases of cervical cancer are estimated in the EU. This tumor is the second leading cause of cancer-related death among AYAs. Almost all cases are attributable to HPV infection, although individual patient susceptibility, immunodepression, and smoking may also play an important role (30). Globally, the incidence is slightly increasing, due to the greater diffusion of oral contraceptives (instead of barrier contraceptives) (31).

Checkmate 358 (32) proved for the first time that immunotherapy might be active in these patients. The study, which involved the use of single-agent nivolumab in recurrent or metastatic cervical, vaginal, or vulvar carcinoma, included women between 28 and 78 years old. Further studies, such as NiCOL trial (enrolling patients aged ≥ 18 years), are now ongoing, exploring the role of immunotherapy also in early stage setting (33).

In 2006, the National Cancer Institute (NCI) Progress Review Group (PRG) on AYA oncology recommended the investigation of potential biologic basis of age-related differences in outcome for AYA cancers. The report of the meeting summarizes the current status of biologic and translational research progress regarding the AYA cancers for which there is the best evidence of a biologic difference between young and elder age groups. These five neoplasms are CRC, breast cancer, acute lymphoblastic leukemia, melanoma, and sarcoma. The report outlines a strategic plan for furthering our basic and clinical knowledge of AYA cancers.

The main differences between AYA and the older population in the aforementioned neoplasms are summarized in **Table 2**.

**Table 2:** Neoplasm main differences in adolescents and young adults *versus* elder patients.

	AYA population	Elder population
**Brain**	Gliomas are the most frequent CNS neoplasms, but adolescents tend to have higher incidence of childhood-related tumors Better survival outcomes in younger patients (4)	Gliomas are the most frequent CNS neoplasms (34)
**Breast**	Often diagnosed later More biologically aggressive tumors (TNBC) (11–13) Lower 5-year survival (14) IMpassion 031 shows a higher rate of pCRs in young patients treated with immunotherapy (35)	Screening programs allow earlier diagnoses (11–13) Higher 5-year survival (14)
**Melanoma**	Women are more affected than men (17) Often genetically predisposed (18) Histologically, a large portion is constituted by spitzoid melanoma (23) High levels of MSH Higher rate of BRAF mutations (>90%) (27, 28) Higher median thickness of the lesion Lymph node involvement is more common Distant recurrence occurs more rarely (16, 24) When treated with immunotherapy, lower rate of colitis and higher rate of hepatitis as irAE When treated with immunotherapy, higher rate of CRs and higher OS (36)	Men are more affected than women (17) UV exposition is the most important risk factor (16) Lower lever of MSH Lower percentage of BRAF mutations (27, 28) Lower median thickness of the lesion Lower rate of lymph nodes involvement Higher probability of distant recurrence (16, 24)

## Safety and Efficacy of Immune Checkpoint Inhibitors in AYA Population

The development of immune checkpoint inhibitors has gone through trials that enrolled mainly adult patients, with a poor representation of AYA patients. Despite these limitations, the available data are promising (37).

In a phase I study, ipilimumab was tested in 33 patients partially compatible with the AYA group (2–21 years old), suffering from melanoma, neuroblastoma, sarcoma, and other solid tumors (38). About 55% of patients developed adverse events (AEs), and 27% developed SAEs. Side effects were similar to those seen in adult patients. Notably, the survival of patients who developed side effects was higher than that of patients who did not.

A recently published study presented the national (Dutch) case history of melanoma in the AYA population by comparing it with the population over 40 years of age (36). A total of 3,985 melanoma patients were analyzed. Of these, 210 fell into the AYA category. The study showed how the frequency of *BRAF* mutation decreases with increasing age, consistent with other previous studies. For what concerns treatment, AYA patients were treated more frequently with combination therapies (anti CTLA-4 plus anti-PD-1). The toxicity profile was slightly different between the AYA group and the over 40 group. Specifically, the AYA group saw more hepatitis during treatment with the immune combination, whereas none in the AYA group experienced colitis on treatment with ipilimumab alone (*vs*. 71/408). The explanation for this has been mainly attributed to the different intestinal flora, which can affect both toxicity and efficacy (39). In particular, it has been demonstrated how the gut microbiome changes according to age, and this affects the population of T helper lymphocytes (CD4), being able to influence the expression of CTLA-4 that is lower in older patients (40). No difference was observed in terms of ORR with the use of anti PD-1, although AYA patients showed a higher rate of CRs than patients over 40 (39% *vs*. 17%). There was no difference in terms of progression-free survival (PFS), while AYAs showed an advantage in terms of overall survival (OS), with 1-year OS of 65% *vs*. 55%, which further improved in the *BRAF*-mutant subgroup.

With regard to breast cancer, the results of IMpassion 031 highlight that efficacy of the combination therapy with atezolizumab is comparable in patients aged less than 40 years with that of elder ones. Moreover, the forest plot seems to show a higher rate of pCR in the younger population.

## Fertility and Pregnancy

### Fertility

With the introduction into clinical practice of immunotherapy (anti-PD-1, anti PD-L1, and anti-CTLA-4) in particular as adjuvant and neoadjuvant therapies, one of the growing perplexities concerns the impact of these therapies on fertility in young patients (41). The NCCN guidelines (42) recommend the use of contraceptive methods for the entire duration of immunotherapy and for at least 5 months after the end of the treatment. Furthermore, most clinical trials require the use of at least two contraceptive methods for the entire time of immunotherapy and for the following 6 months for all patients enrolled and treated in reproductive age. However, there is currently a lack of data to support these recommendations and, at the same time, the indications could underestimate the risks, since it is well known that immune checkpoints can occupy their receptors for a period of time that can reach 30 months (43). The lack of data on fertility implies the need to amplify research efforts in this field.

A group of particular interest, for example, is represented by those patients who have suspended/discontinued immunotherapy or have terminated it, as per clinical practice (e.g., adjuvant treatment in melanoma). In this group, it would be appropriate to measure parameters commonly used as surrogates of fertility, such as the rate of spontaneous abortions, stillbirths, and circulating hormones such as Anti-Mullerian Hormone (AMH).

Anti CTLA-4 and anti PD-1 have an uncertain impact on fertility, but they seem to be able to act directly on oogenesis and spermatogenesis and can affect conception in various ways, including endocrinological dysfunctions that can directly or indirectly afflict the reproductive organs. Hypothyroidism, but especially pituitary gland dysfunctions, can negatively affect ovarian and testicular function. The destruction of these axes can in fact also lead to severe consequences such as premature menopause, the decrease of testosterone levels and the consequent erectile dysfunction, and the reduction in sperm production. In addition to what has been said, premenopausal women seem to be more affected by immune-related adverse events, thus placing this group of patients in a higher risk range of infertility (44).

### Pregnancy

Cancer concomitant with pregnancy represents an important clinical challenge, with clinical and obstetric implications not yet fully described. Recently, a meta-analysis by Lambertini et al. showed that pregnancy in breast cancer survivors is safe and the pregnancy desire should be considered with the due importance in the patient’s care story (45). The impact of immunotherapeutic agents has so far been studied to little extent in this population, first because pregnant women are excluded from all clinical trials and, second, because this event is more compatible with early stage, and only recently has immunotherapy found a place in the neo/adjuvant setting.

Regarding the incidence, in 2009, in a retrospective Norwegian cohort study, an incidence of one cancer diagnosed in every 2,000 pregnancies is reported (46), while the incidence in an Australian study was found to be 137.3 per 100,000 (47).

Although some differences occur among studies, the most frequently diagnosed tumors during pregnancy are breast cancer, malignant melanoma, gynecological tumors (especially of the uterine cervix), as well as tumors of the thyroid and lymphohemopoietic system (48). In the 2 years after giving birth, the most frequently diagnosed cancer was breast cancer, followed by melanoma and cervical cancer (48).

Much interest is also aroused in the outcome of patients diagnosed with cancer during pregnancy, and there are differences based on the type of cancer considered. An increased risk of death is observed for melanoma diagnosed during pregnancy, but not if the diagnosis is made during breastfeeding; on the contrary, the risk of death from breast cancer increases if the diagnosis is made during breastfeeding. One explanation could be the diagnostic delay caused by the misinterpretation of signs and symptoms of cancer attributed to normal changes caused by pregnancy. This is the case, for example, of malignant melanoma, the characteristics of which are mistaken for skin changes that might occur during pregnancy (49). Moreover, mammograms during breastfeeding are difficult to interpret. Finally, both clinicians and patients tend to postpone diagnostic tests in this time interval.

The prognosis of ovarian cancer diagnosed during breastfeeding is worse, with an increased risk of death, while the risk is lower if the diagnosis is made during pregnancy, probably due to the pregnancy follow-up protocol that allows for an earlier diagnosis of malignant lesions and due to tumors that are diagnosed during cesarean delivery (46).

Although preclinical evidence on non-human models demonstrates that immunotherapy is able to lead to an unfavorable pregnancy outcome, real-world reports do not seem to demonstrate an impact of these drugs on the health of the fetus, either in monotherapy or in combination. However, cases of intrauterine growth retardation are reported by several published cases. Nevertheless, it should be noted that the follow-ups available on the state of growth and wellbeing of children are rather short. Considering the scarcity of evidence and the difficulty, even the ethics, of carrying out prospective immunotherapy studies in pregnant women, the analysis of published case reports plays a fundamental role in order to better understand the interactions between these drugs and this particular subpopulation of patients.

#### Pregnancy and Anti-CTLA-4

Currently, ipilimumab has been categorized in group C by the FDA, due to the unsafe role of CTLA-4 in maternal fetal immunotolerance. Preclinical studies of ipilimumab have shown changes in the connective tissue of the ovary, without other histological differences, and a decrease in the weight of the testicles, in the absence of alterations in sperm production (50).

In animal models, ipilimumab was administered at 2.6–7.2 times the recommended dose of 3 mg/kg, and it induced an increased rate of third-trimester miscarriages, stillbirth and perinatal mortality, premature births, and low birth weight. The FDA package insert of ipilimumab suggests that these effects could be more severe if the drug is administered in the second and third trimester of pregnancy. In pregnant women, however, we do not know exactly how ipilimumab affects pregnancy or the product of conception. The US FDA Adverse Event Reporting System database in 2017 contained seven cases of ipilimumab exposure during pregnancy (51). The outcomes of these pregnancies were a spontaneous abortion in the case of one patient who also received dabrafenib, a stillborn fetus, an ectopic pregnancy, two pregnancies that ended with therapeutic abortions, and two pregnancies whose outcomes have not been reported. Still in 2017, the database did not report any cases of fetal malformations. Finally, a case report described the results of a further pregnancy in a 31-year-old patient with metastatic melanoma. Pregnancy was discovered during the third cycle of ipilimumab, when the patient was already in the sixth week of gestation. In agreement with her doctor, and after rejecting the hypothesis of an abortion, the fourth administration of ipilimumab was performed. The patient showed no autoimmune adverse events during pregnancy, with the exception of a G1 diarrhea. Fortunately, the pregnancy outcome was favorable, and the patient gave birth to a healthy baby, in the absence of apparent malformations. There is no long-term follow-up of the child’s health.

#### Pregnancy and Anti-PD-1

A paper published in 2016 provided the main preclinical data regarding the possible reproductive toxicity of anti PD-1 agents (52). The PD-1/PD-L1 axis is indeed a fundamental pathway in maintaining maternal fetal immunotolerance, and its alteration potentially seems to be able to reduce or even block it. However, if, on the one hand, the therapeutic effects of anti PD-1 antibodies have been demonstrated in mouse models and successfully translated into human experimentation, on the other hand, the potential adverse events on fertility have been studied in animal models but not in human subjects. PD-L1 expression was in fact observed in mouse models, at the utero-placental interface in case of allogenic conception: in these models, the use of antibodies against PD-L1 led to a significant increase in the number of miscarriages (86%) (53), with an effect that seemed to depend on the presence or absence of Treg lymphocytes. Indeed, the transfusion of regulatory T lymphocytes from healthy animals to treated animals was able to restore physiological immunotolerance (54). In the surviving fetuses, however, no malformations or deficit of fetal development was recorded at birth.

Some cases of infertility with atezolizumab have been reported in animal models, with menstrual changes and lack of new corpus luteum formation in the ovaries. However, the dosage at which these side effects were observed was six times the dose used in clinical practice, and all events proved to be reversible. No side effects were found in the male subjects (from Atezolizumab. Package insert. Genentech, Inc.; 2019).

The treatment with pembrolizumab (and similarly nivolumab and durvalumab) in animal models (monkeys) did not show direct effects on the reproductive organs, although many animals were not sexually mature (52). In the light of the abovementioned data, the anti PD-1 agents have been categorized in category D in relation to the risk of pregnancy by the FDA.

In clinical practice, experiences are limited. A recently published case report (55) presents the case of a 39-year-old woman with metastatic uveal melanoma, treated during the first 6 weeks of a twin pregnancy, with nivolumab alone. After the development and resolution of an autoimmune hepatitis for which the patient had received azathioprine, treatment with nivolumab was suspended upon discovery of pregnancy. Despite the suspension, the pregnancy was complicated by the development of a HELLP (Hemolysis Elevated Liver enzymes Low Platelet count) syndrome at the 27th week, resulting in the induction of childbirth at the 30th week. The twins had already shown growth disparity by week 24; at birth, one twin was smaller than the other, and the smaller one presented agenesis of one hand (interpreted by the gynecologist as a result of strangulation with the umbilical cord). At postpartum restaging, the patient was NED (No Evidence of Disease).

In another short communication published in 2019 (56), the case of a 32-year-old woman with stage IV melanoma (lymph nodes and liver) diagnosed in 2016 is presented. The patient had an unspecified history of infertility, with unsuccessful attempts to have children that lasted for about 10 years. The patient received ipilimumab plus nivolumab for 4 cycles, followed by maintenance with nivolumab alone. After 10 months of treatment, the patient underwent a complete metabolic response, accompanied by an increase in GGT, rash, vitiligo, and hypophysitis with compromise of the pituitary–adrenal axis. After 14 months of treatment, a 7-week pregnancy was diagnosed. In the light of the pregnancy, and the patient’s desire to continue with it, nivolumab was discontinued. The patient gave birth at 33 weeks, *via* caesarean section, to a premature baby, who suffered from congenital hypothyroidism. There was no metastasis to the placenta.

As regards the combination of anti-CTLA-4 and anti-PD/L-1, two case reports could give us information of their impact on pregnancy. The first one (57), published in 2018, concerns a 34-year-old woman affected by stage IV cutaneous melanoma, with metastasis in lymph nodes, lung, liver, and bone diagnosed in the seventh week of pregnancy. The patient started ipilimumab in combination with nivolumab, and she received four cycles of treatment in the absence of toxicity. The treatment was subsequently suspended due to the development of autoimmune hepatitis during maintenance therapy with nivolumab alone. The birth took place in October 2016, with a caesarean section: the baby was premature but did not show signs of developmental deficit.

The second case report, also published in 2018 (58), concerns a 34-year-old patient with stage IV melanoma with pleural, lung, bone, liver, and spleen localizations, diagnosed in 2017,during pregnancy. The patient started the combination of ipilimumab and nivolumab. The therapy was suspended after two cycles due to a marked worsening of the clinical conditions. It was therefore proposed to induce pulmonary maturation of the fetus and to subsequently perform a caesarean delivery. The patient died the day after giving birth to a severely premature baby, but with no signs of malformations or developmental deficits.

The third case regards a twin pregnancy. The pregnancy ended with the birth of two healthy babies, characterized only by low weight at birth (59).

## Long-Term Adverse Events

In addition to what has been reported, other toxicities may be of particular interest in young patients and, as it happens for endocrinological changes, may lead to long-term effects. It is well known that response to immunotherapeutic agents may be durable and may last even beyond the end of the treatment. Therefore, it is reasonable to believe that immune-related toxicities may develop independently from dose and duration of treatment. Indeed, occupancy of PD-1 on T cells is estimated to plateau approximately 80–90 days after one single dose of nivolumab and be still approximately 40% 8 months after three doses (60).

Reports of AEs in clinical trials are crucial for the definition of a risk/benefit ratio and for the correct interpretation of the clinical benefit of a specific treatment in a specific setting. In this regard, immunotherapy pivotal trials often reported adverse events in an uncomplete and suboptimal way (61). For instance, clinical trial publications are accurate about the course of the treatment with immunotherapy, but very rarely about when it was discontinued. Moreover, follow-up for adverse events is often limited in time, so that, even if delayed toxicities are reported in literature as a possible entity, they have been rarely described before 2018 and they are generally underreported in immunotherapy registering trial safety assessment.

Couey et al. reviewed immunotherapy clinical trials published between 2008 and 2018. Through this research, they individuated a median safety reporting window of 90 days. These data were used as a cutoff to define Delayed Immune-Related Events (DIRE) in a subsequent literature research: 21 cases were found, with more than half reported since 2018. The target organs and systems were multiple: endocrine (7 patients), cutaneous (5), neurologic (5), pulmonary (3), cardiac (3), gastrointestinal (2), rheumatologic (1), and ophthalmologic (1) (62).

Moreover, the case series is enriched by two other cases that occurred at the institution of the authors, both characterized by initial misdiagnosis and subsequent erroneous management.

To note, more than half of the cases individuated in the review of literature developed after a brief course of immunotherapy (≤4 doses) (62).

Ghisoni et al. recently analyzed immunotherapy pivotal trials published until 2019 and real-world data of lung cancer and melanoma patients treated at their institution (University Hospital of Lausanne) between 2011 and 2019. The authors aimed to highlight how report and description of late-onset AEs is often underreported in the literature. Indeed, 90% (56/62) of the publications of pivotal trials leading to approval of immunotherapeutic agents did not specify the percentage of patients still affected by toxicities at data cutoff (63).

These considerations are particularly important since the use of immunotherapy is gradually expanding in adjuvant and neoadjuvant setting, which implicate short courses of treatment and the possibility of a complete remission of the neoplasm. In this scenario, the eventuality of long-term or late-onset toxicities, in patients who may be potentially healed from cancer, must be carefully considered.

Reports of delayed immune-related adverse events in literature have become more common since 2018, suggesting that physicians are more and more conscious of these entities. However, longer follow-up in clinical trials and real-world studies are warranted in order to identify and collect late onset or toxicities that last beyond the window of the treatment. In 2020, an Italian multicenter retrospective study evaluated the impact of late immune-related adverse events (IrAEs) on patients treated for advanced disease with ICIs. They discriminated early IrAEs (if previous than 12 months from starting immunotherapy) and late IrAEs (>12 months from starting immunotherapy). All the AEs were more frequent in the first 12 months. The most frequent IrAEs were cutaneous (11.5%), rheumatologic (6.7%), endocrine (4.6%), and gastrointestinal (4.1%) ones. Most of these effects did not need any intervention but only supportive care (44.7%), 32.5% started steroid without discontinuation of treatment, and 11.4% had a temporary interruption with immunotherapy and have been treated with corticosteroids. Curiously, only in the late IrAEs group did 11.4% of patients start corticosteroid with permanent ICIs discontinuation, while patients with early IrAEs never discontinued immunotherapy.

## Discussion

Data relating to AYA patients are scarce due to the lack of dedicated studies and poor enrollment in clinical trials. The advent of immunotherapy has completely changed the therapeutic approach in various cancers in advanced stages and now these changes are increasingly moving to early settings. The prospect of treating patients potentially cured or curable from cancer with immunotherapy opens up new scenarios and new challenges of primary importance. Data relating to AYA patients are the only ones that allow us to not only address some typical aspects of young age such as fertility and pregnancy but also understand how other IrAEs can affect patient’s life in the long term, thus giving us information that are useful for the treatment of elder patients, too. As abovementioned, most frequent diagnosed cancers in the AYA subgroup are breast cancer, melanoma, gliomas, testicular cancers in male patients, and thyroid cancers (1). Recently, immunotherapy has become standard of care in the adjuvant setting for melanoma, and new data regarding the neoadjuvant setting are emerging (64). Furthermore, in TNBC, immunotherapy showed to increase complete pathological responses (15): indeed, both Keynote 522 and IMpassion 031 (35) demonstrated that immunotherapy could gain 13%–17% of pathological response more than standard chemotherapy. Encouraging data on the impact of immunotherapy were also seen in the neoadjuvant treatment of gliomas (6).

Recently, immunotherapy has also shown to increase efficacy in adjuvant setting in tumors of the esophagus and gastroesophageal junction (65). All these data that are rapidly being obtained show us how immunotherapy is a strategy not only intended for patients with advanced and incurable disease, but it is increasingly gaining its place in the treatment of patients who might survive cancer. Neo/adjuvant chemotherapy has taught us to face some particular situations such as reduction of fertility or management of pregnancy concomitant with diagnosis of cancer, as well as management of other long-term side effects such as peripheral neuropathies (taxanes, platinum, etc.), and cardiac dysfunction (trastuzumab, anthracyclines, etc.).

The impact of chemotherapy on fertility and pregnancy has been studied for years in patients affected by breast cancer, and fertility preservation strategies, such as concomitant administration of LH-RH analogous, cryopreservation of embryos/oocytes, have been proposed for these patients (66). In the case of breast cancer patients, the strategies are already defined since chemotherapy is the therapeutic backbone for these patients. The experience of other tumors is different. Until recently, immunotherapy was intended only for the setting of advanced disease where, unfortunately, strategies for the preservation of female or male fertility lose their meaning due to poor prognosis of these patients *a priori*. Currently, with the employment of immunotherapy in the adjuvant setting, the almost total lack of data on the impact of immunotherapy on fertility must be considered. This implies that it is unclear whether fertility preservation strategies, which are normally adopted for patients treated with chemotherapy in early stage tumors, are indicated in these cases, too (67).

The data collected in this review, albeit limited, reassure us in proposing immunotherapy as an effective treatment in early settings. Surely, treatment with immunotherapy in pregnancy requires a very accurate multidisciplinary discussion, first because available data are derived only from case reports and, secondly, because immunotherapy, and especially anti-PD-1 agents, seems to have a non-negligible impact on gestation, having the potential to break the maternal–placental immunotolerance, which is largely entrusted to the PD-1/PD-L1 axis. Indeed, preliminary data and the few clinical experiences seem to show minor consequences in patients treated with anti-CTLA-4 agents. Unfortunately, collecting systematic data in these patients is very difficult also due to the ethical implications that this entails. Thus, we can only discuss case reports, which, to date, are the only way we have to assess the risk of complications for the mother and/or fetus.

For all these reasons, a pregnant woman who has to face an oncological disease with a potential benefit deriving from immunotherapy must know that there is a risk of abortion and premature births. However, there is no evidence that immunotherapy can have a teratogenic effect in live births. Finally, we must take into account the most known toxicities of immunotherapy such as endocrinological, gastrointestinal, dermatological, and rheumatological ones. In the treatment of advanced malignancies, we have learned to recognize and manage these toxicities. Some of these, such as the dermatological and gastrointestinal ones, may be completely resolved. On the contrary, endocrinological toxicities, such us hypothyroidism following thyroiditis, are often permanent and must be treated with continuous replacement therapy. Rheumatological toxicities are not always completely resolved and sometimes definitive joint and functional limitations arise.

The evolution of adjuvant and neoadjuvant treatments improves the chances of curing cancer patients, and the addition of immunotherapy makes an important contribution to this. At the same time, it is more and more necessary to study and collect long-term data not only regarding efficacy, but also toxicity, in order to provide, on the one hand, deeper information for the patient and, on the other hand, a targeted search for strategies to improve the quality of life of cancer survivors. In particular, it will be important to collect the data of these patients in daily clinical practice with *ad hoc* observational studies to evaluate the most specific side effects for this age group such as the effects on fertility and pregnancy.

## Author Contributions

GR and ET contributed to conception and design of the review. EC, SS, and LZ organized the data and reviewed the literature. LZ, EC, GR, ET, CG, and FS wrote sections of the manuscript. All authors contributed to the article and approved the submitted version.

## Conflict of Interest

CG received honoraria from Astra Zeneca, Bristol-Myers-Squibb, Merck-Sharp-Dohme, Roche, and Boehringer-Ingelheim.

The remaining authors declare that the research was conducted in the absence of any commercial or financial relationships that could be construed as a potential conflict of interest.

## Publisher’s Note

All claims expressed in this article are solely those of the authors and do not necessarily represent those of their affiliated organizations, or those of the publisher, the editors and the reviewers. Any product that may be evaluated in this article, or claim that may be made by its manufacturer, is not guaranteed or endorsed by the publisher.
